# Genome Sequence of Mycobacteriophage ErnieJ

**DOI:** 10.1128/genomeA.00873-16

**Published:** 2016-11-23

**Authors:** Courtney J. Robinson, Laricca Y. London-Thomas, Leon A. Dickson, Tiffany A. Clinton, Hana Baig, Maude Bute, Mohammed Fahad, Kanhai Farrakhan, Neshaun Grady, Nicholas E. Guthrie, Ruoa Hafid, Jayla Harvey, Kellie Hunnicutt, Victoria L. Larsen, Taashaylaray McDuffie, Earyn N. McGee, Jillian Y. Pailin, Bria Peacock, Antolice Thomas, Winston A. Anderson

**Affiliations:** a Department of Biology, Howard University, Washington, District of Columbia, USA; b Department of Microbiology, Howard University, Washington, District of Columbia, USA

## Abstract

ErnieJ, a cluster C mycobacteriophage that infects *Mycobacterium smegmatis* mc*^2^*155, was recovered from soil in Washington, DC. Its genome is 153,243 bp in size and encodes 227 predicted proteins, 30 tRNAs, and one transfer-messenger RNA (tmRNA). Ten percent of the predicted proteins have homologs in phages that infect nonmycobacterial *Actinobacteria*.

## GENOME ANNOUNCEMENT

Bacteriophages have been instrumental in deciphering fundamental biological processes (1). Additionally, the potential for phages to be used therapeutically against bacterial pathogens has been long recognized and is reemerging as a popular research area (2). These viruses are genetically diverse, and questions regarding the breadth of genetic diversity as it relates to phages that infect a single host are starting to be answered (3).

Mycobacteriophage ErnieJ, named after developmental biologist Ernest Everett Just, was isolated in 2012 from soil collected on the campus of Howard University in Washington, DC, via enrichment at 37°C using *Mycobacterium smegmatis* mc^2^155 as a host. Electron microscopy revealed that ErnieJ is a myovirus. Purification by streaking was followed by generation of a high-titer lysate and DNA isolation. The genome was sequenced using 454 pyrosequencing at the Virginia Commonwealth University Nucleic Acids Research Facilities. Reads were assembled using Newbler and Consed into a single major contig, with a coverage of 120-fold. The circularly permutated genome is 153,243 bp and has a G+C content of 64.7%. Genomic analysis showed that ErnieJ is a member of mycobacteriophage cluster C, subcluster C1 (3). These lytic phages contain the largest mycobacteriophage genomes, with an average size of 155,694 bp. Upon comparison to genomes in the Actinobacteriophage Database (phagesdb.org), it was revealed that ErnieJ is most similar to ChickenPhender. The genomes share 99% nucleotide identity across 97% of the ErnieJ genome. A comparison to genomes in GenBank revealed highest similarity to phage Sebata, with 98% nucleotide identity with 93% coverage.

The ErnieJ genome was annotated using DNA Master (http://cobamide2.bio.pitt.edu/). Putative protein-coding regions and tRNAs were identified using Glimmer (4), GeneMark (5), Aragorn (6), and tRNAscan-SE (7). Predicted gene functions were assigned using BLASTp (8), HHpred (9), and Phamerator (10). Using combined evidence from these sources, the start sites identified by autoannotation in DNA Master were manually adjusted when necessary. The genome contains 30 tRNAs, one transfer-messenger RNA (tmRNA), and 227 predicted protein-coding genes, 48 of which could be assigned functions. Functional assignments for predicted proteins included virion structure and assembly, nucleotide and protein synthesis, helicase, and endonuclease, among others. As expected, a programmed translational frameshift was detected in the tail assembly chaperone gene upstream of the tape measure gene. While 67% of the predicted proteins are most similar to cluster C mycobacteriophages, 14.7% also share similarity with those from other mycobacteriophages. Another 14.7% group with genes from various mycobacteriophage clusters and phages that infect different hosts, while eight genes were only homologous to other cluster C genes and genes from phages that infect nonmycobacterial hosts. For example, one of the latter genes is predicted to encode a thymidylate synthase that has homologs in other cluster C mycobacteriophages and phages that infect *Streptomyces*, *Rhodococcus*, and *Gordonia*. Interestingly, ErnieJ also encodes three glycosyltransferases, one of which is only found in cluster C phages, another which is present in multiple clusters, and one that is present in other clusters and in a *Gordonia* phage.

### Accession number(s).

The ErnieJ genome sequence was submitted to GenBank under accession number KT365400.
